# PERSISTENCE OF ACCELERATED VASCULAR AGING FOLLOWING THERAPY IN OLDER CANCER SURVIVORS

**DOI:** 10.1093/geroni/igz038.3467

**Published:** 2019-11-08

**Authors:** Barbara W Carlson, Melissa Craft, Rachel Funk-Lawler, Karla Keepper, Wajeeha Razaq, Anna Csiszar

**Affiliations:** 1 Fran and Earl Ziegler College of Nursing, The University of Oklahoma Health Sciences Center, Oklahoma City, Oklahoma, United States; 2 Department of Psychiatry, University of Oklahoma Health Sciences Center, Oklahoma City, Oklahoma, United States; 3 Stephenson Cancer Center, The University of Oklahoma Health Sciences Center, Oklahoma City, Oklahoma, United States; 4 Reynolds Oklahoma Center on Aging, The University of Oklahoma Health Sciences Center, Oklahoma City, Oklahoma, United States

## Abstract

Chemotherapy destroys cells indiscriminantly and releases proinflammatory factors into the bloodstream that can adhere to endothelial cells (ECs); resulting in phenotypical changes consistent with “accelerated vascular aging”. This pilot study examined associations between markers of EC integrity, vascular aging, and cognition in 15 female breast cancer survivors 12–18 months after chemotherapy (median age: 57 years) and 2 non-cancer controls (58/59 years). EC integrity was evaluated using frequency-dependent electrical impedance (Z4000Hz) and levels of apoptosis (caspase-3/7), inflammation (NFkB activation), and oxidative stress (NRF2 activation) in cultured human endothelial cells (EC) exposed to the subject’s serum. Vascular aging was characterized by low serum insulin growth factor ([IGF1a] <85mg/dL) and laterality in cerebral oxygenation (Lat-FLOX), an in-vivo marker of altered cerebral blood flow (near-infrared spectroscopy). Z4000Hz were higher in cells treated with serum from survivors (520-1100 ohms) than controls (400-450 ohms). Higher Z4000Hz was associated with higher amounts of EC oxidative stress (rNRF2= -.55), inflammation (rNFkB= .43), apoptosis (rcapsase=.76), and Lat-FLOX (r=.39). Higher Z4000Hz (r= -.48) and Lat-FLOX (r= -.66) were associated with lower cognitive function, as measured by the Montreal Cognitive Assessment (MOCA). At lower Z4000Hz, combined Lat-FLOX and low IGF1a characterized persons with cognitive impairment (MOCA < 26). These findings suggest that proinflammatory factors related to cancer and/or its treatment persist for months following active treatment. Cognitive symptoms occur when EC damage results in alterations in cerebral blood flow. With depletion of growth factors, like IGF-1, these symptoms may occur at lower levels of EC damage.

